# Lactopeptine

**Published:** 1886-02

**Authors:** 


LACTOPEPTINE.

We have used this article for some time in cases of indigestion, and can recommend it as a valuable remedy. Being a compound of the five active agents which are contained in the process of digestion, it cannot fail to aid the system in preparing the food for assimilation. It is an invaluable remedy in the summer diarrhœa of children. Owing to its great impairment of the vital forces, and feeble powers of the digestive tract, food frequently irritates and increases the difficulty. For such cases we learn of no agent in the Materia Medica as reliable as Lactopeptine.—*Cal. Med. Jour.*

